# Appetite Assessment of Hospitalized Cancer Patients in Brazil – A Validation Study

**DOI:** 10.6061/clinics/2019/e1257

**Published:** 2019-10-09

**Authors:** Gislaine Aparecida Ozório, Maria Manuela Ferreira Alves de Almeida, Sheilla de Oliveira Faria, Thais de Campos Cardenas, Dan Linetzky Waitzberg

**Affiliations:** Servico de Nutricao e Dietetica, Instituto do Cancer do Estado de Sao Paulo (ICESP), Hospital das Clinicas HCFMUSP, Faculdade de Medicina, Universidade de Sao Paulo, Sao Paulo, SP, BR; Departamento de Medicina Preventiva, Faculdade de Medicina FMUSP, Universidade de Sao Paulo, Sao Paulo, SP, BR; Nutricao e Dietetica, Instituto Brasileiro de Controle do Cancer, Sao Paulo, SP, BR; Departamento de Gastroenterologia, LIM-35, Faculdade de Medicina FMUSP, Universidade de Sao Paulo, So Paulo, SP, BR

**Keywords:** Cancer, Appetite, Malnutrition, Visual Analog Scale

## Abstract

**OBJECTIVES:**

Appetite loss, a common symptom in cancer patients, contributes to worsened nutritional status. A validated specific tool to assess appetite is clinically useful for diagnosing and identifying symptoms and signs that could be reversed with nutritional and pharmacological therapies. The aim of this study is to produce a Brazilian Portuguese version of the Hill and Blundell visual analog scale (VAS) for appetite and investigate its validity among hospitalized cancer patients.

**METHODS:**

The original English VAS version was translated into Brazilian Portuguese in full accordance with the guidelines in the literature and adapted to the Brazilian context by conducting interviews and meetings with an expert committee until the final version was reached. Afterwards, the version was validated in hospitalized cancer patients in a cross-sectional study at São Paulo Cancer Institute (ICESP), where the relationships between breakfast intake (rest-ingestion index) and VAS were compared. The Spearman test was used to verify the correlation between the rest-ingestion index and the VAS ratings.

**RESULTS:**

Sixty-four patients with a mean age of 56.1 (±12.3) years answered the Portuguese VAS version, and their breakfast intake was evaluated. The mean rest-ingestion index was 18.8%. The correlations between the rest-ingestion index (food acceptance) and three questions of the Portuguese visual analog scale version were inverse and significant: first question (ρ -0.3028 *p*=0.0046), second question (ρ -0.2317 *p*=0.0319) and third question (ρ -0.3049 *p*=0.0043).

**CONCLUSION:**

The “Appetite Assessment Scale of Brazilian Oncology Patients” is a valid instrument to assess appetite in hospitalized cancer patients in Brazil.

## INTRODUCTION

Loss of appetite is common in cancer patients and can occur, among other factors, due to changes in the central nervous system and peripheral neurohormonal signs that govern appetite (1,2).

The incidence rates in patients with advanced cancer can range from 39.0% to 81.5% for weight loss and 30.0% to 80.0% for anorexia (3). Lack of appetite was reported by 80.0% of patients in palliative care (4). This broad variation reflects conditioning factors such as distinct assessment patterns, selection of different clinical populations (stages of cancer) and inconsistent methodologies including retrospective analysis of medical records, cross-sectional assessments and longitudinal designs.

There is an association between lack of appetite and radiotherapy. In patients with head and neck cancer, the amount of irradiation was related to the worsening of appetite. At 20 Gy of radiation, a lack of appetite was associated with lower sensitivity to taste. With a higher frequency (50 Gy), lack of appetite was associated with oral mucositis, dry mouth, low saliva production in the morning, reduction in taste sensitivity, analgesic use and frequency of oral care (5).

Loss of appetite was present in 64.0% of patients with gastroesophageal cancer. The highest intensity of appetite loss was associated with tumor size, staging, the impossibility of surgical treatment, weight loss, and dysphagia (6). Similarly, in esophageal cancer patients, there is an association between loss of appetite and worse survival rates (7,8).

Difficulties in the diagnosis of anorexia and the identification of anorexia-cachexia syndrome (SAC) lead to detrimental consequences for patients’ nutritional status (9). SAC is characterized by weight loss (5.0% or greater) and inflammatory and metabolic abnormalities that include changes in the intermediary metabolism of carbohydrates, lipids and proteins (10).

Due to the high prevalence of appetite loss in cancer patients, it is of interest to have a specific tool to assess appetite and diagnose and identify the symptoms and signs that can be reversed with nutritional and pharmacological therapeutic strategies. The proper detection of appetite loss helps in the early indication of specific dietary and pharmacological measures (11). However, the measurement of appetite is challenging because it is subjective and experienced differently by each individual.

In clinical studies, appetite is often assessed using visual analog scales (VASs), which record subjective sensations, such as hunger and fullness. The use of VASs has been particularly popular for the assessment of pain and appetite (12).

According to studies that have evaluated appetite, VASs are sensitive to several types of experimental manipulations, including changes in diet composition (13,14), changes in energy intake (15) and administration of drugs that stimulate or inhibit appetite (16).

In Brazil, there are no records of validated scales for the evaluation of appetite among cancer patients. A widely used validated version is beneficial for conducting clinical and epidemiological research on this topic and for use in clinical practice. Thus, this study was performed with the objective of producing a Brazilian Portuguese version of the Hill and Blundell VAS for the assessment of appetite and to investigate its validity.

## METHODS

This study was conducted at São Paulo Cancer Institute (ICESP) in Brazil from January to September 2013. The project was approved by the Research Ethics Committee of the Faculty of Medicine of São Paulo University (CEP-FMUSP) (protocol n° 6639).

The present study consisted of two parts: translation and validation.

The VAS developed by Hill & Blundell (1982) was chosen because it is a nonextensive scale, easy to understand, complete and used/validated in other languages (12,14,). It is a VAS with five questions. On each 100-mm line, an appetite sensation is paired with the opposing sensation (for example, ‘hungry’ and ‘not hungry’).

### Translation

Four independent translators (native Brazilians), including three health professionals (nutritionists) and one English teacher, translated the VAS from English into Brazilian Portuguese. A synthesis of the translations was agreed upon by consensus between translators (V1). Eleven participants were interviewed to verify their comprehension of V1. Those patients completed the scale with assistance: questions were read aloud by the interviewer. Participants were asked about their understanding of the scale and were invited to offer suggestions that could clarify its meaning. An expert committee reviewed all of the reports and reached consensus on a new version (V2).

Four independent native English speakers who fluently speak Brazilian Portuguese created a back translation into English. The translators were blinded to the original version. The back translations were synthesized by consensus between translators (V3) and were compared with the original version of the scale to verify the reliability. The main authors (Hill and Blundell) examined this combined version and gave consent for its use.

**Figure 1:**
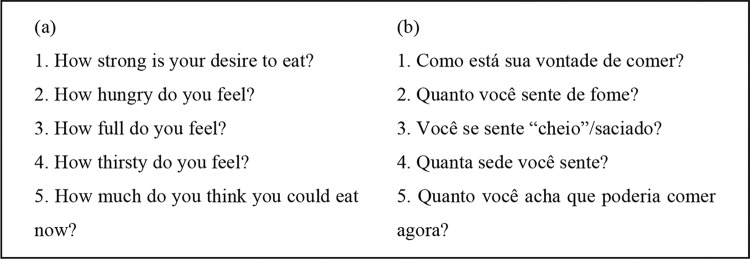
(a) Original visual analog scale; (b) Appetite Assessment Scale of Brazilian Oncology Patients “EAA-BR”.

Finally, based on the consensus of the researchers and a multiprofessional nutritional therapy team from ICESP, the final version in Brazilian Portuguese was created (Version 4 - Appetite Assessment Scale of Brazilian Oncology Patients “EAA-BR”-“Escala Avaliação Apetite em Pacientes Oncológicos Brasileiros”) (Figure 1).

### Validation of the EAA-BR

To perform the validation of the EAA-BR, a preliminary cross-sectional study was performed in clinically and surgically hospitalized patients from August to September 2013. The inclusion criteria were 18 years of age or older, conscious and guided state according to the nursing team (Glasgow 15, Richmond Agitation-Sedation Scale (RASS) 0 and negative Confusion Assessment Method (CAM)), consumption of an exclusively oral diet (normal or soft) and ability to provide meaningful informed consent.

Exclusion criteria were the use of enteral or parenteral nutrition, reduced level of consciousness or severe psychiatric illness, aerosol or contact isolations, inability to perceive taste after partial or total glossectomy, presence of brain tumor or cranial trauma, severe pain that could interfere with food intake, dysphagia and inability to understand the questions of the VAS.

Data on age, sex, oncological diagnosis, reason and length of hospital stay, body mass index (BMI) and presence of weight loss ≥5% in the last 3 months (prior to admission) were collected from the electronic medical chart. The use of corticosteroids within two days prior to the interview date was verified.

Nutritional Risk Screening - 2002 (NRS-2002) (22) and Global Subjective Assessment (ASG) (23) were used to assess nutritional risk and nutritional status, respectively. BMI was classified according to the World Health Organization proposal (24) for individuals aged up to 60 years and according to the Pan American Health Organization (25) for individuals aged 60 years and over.

To validate the EAA-BR, it was compared in relation to the amount of food effectively ingested by the patient during breakfast. The application of the appetite scale (EAA-BR) was performed before the meal, and the total amount of food consumed at the test meal was measured by weighing food items before and after eating to the nearest 1 g (Dayhome^®^, Sao Paulo, SP). The rest-ingestion index was calculated according to the following formula: rest-ingestion index (%) = (quantity rejected (g) / quantity offered (g)) x 100 (26).

For descriptive analysis, quantitative variables were presented as the mean and standard deviation. The Shapiro-Wilk test was used to assess normality. For the variables that were not normally distributed, the Spearman test was used to verify the correlation between the rest-ingestion index and the EAA-BR ratings. Significance was accepted at the level of *p*<0.05. Those older than 60 years were considered elderly.

## RESULTS

Sixty-four patients were evaluated. The mean age was 56.1 (±12.3) years; 52.3% were females. Gynecological cancer (29.7%) was the most prevalent, followed by malignant gastrointestinal tumors (21.9%) and urological tumors (21.9%). The majority (88.4%) either received or were receiving some type of cancer treatment during data collection (Table 1).

The use of corticosteroids was present in 31.3% of patients. One-fourth of the patients (25.6%) had significant weight loss (≥5%) in the last 3 months prior to admission. Cancer treatment was the main reason for hospital admission, with surgical reasons being the most prevalent (80.7%). The second reason for hospitalization was related to the presence of symptoms, including headache, dyspnea, paresthesia, nausea, vomiting, hyperkalemia, diarrhea, weakness, vaginal bleeding, hypoxemia/hemoptysis, mental confusion, dizziness, among others. The mean hospital stay until the interview was 5.7±6.5 days, with a total hospitalization time of 11.2±11.5 days (Table 1).

The mean BMI was 26.6±7.0 kg/m^2^, and 51.6% were classified as eutrophic (Table 2).

The mean rest-ingestion index was 18.8%. Among the patients who consumed less than 50% of the food offered, the most reported reasons for not accepting were lack of appetite (17%), nausea/vomiting (15%) and amount of food offered (15%).

The correlation between the rest-ingestion index and food acceptance was inverse and significant for three questions: the first question (ρ -0.3028 *p*=0.0046), the second question (ρ -0.2317 *p*=0.0319) and the third question (ρ -0.3049 *p*=0.0043) (Table 3).

## DISCUSSION

There were no validated questionnaires for the assessment of appetite in Brazilian Portuguese. This study demonstrated that the EAA-BR is a valid and acceptable measure for assessing appetite in Brazilian cancer patients.

The rest-intake index revealed different results from those of the study by Ferreira et al. (26). According to these authors, the rest-lunch intake index was 37.0% and significantly higher among the undernourished patients, as detected by the Subjective Global Assessment (PG-SGA) (*p*=0.004). A likely explanation is that, at breakfast, patients have greater food acceptance because of night fasting. In addition, breakfast foods, in general, do not produce odors, which could cause nausea (26).

As reported by Ferreira et al., an excessive amount of food offered was one of the reasons for not accepting the meal (26). In addition, they described lack of appetite (26%), lack of flavor (40.0%) and monotony of preparations (33.0%) as reasons for not accepting the diet. Decreased food intake may also occur due to changes in general habits, hospital environment and dissatisfaction with dietary preparations. Food offered in hospitals is commonly judged as insipid, tasteless, cold, and served early. Patients hospitalized at ICESP (unpublished data) routinely evaluate the quality of the meals offered as excellent in terms of temperature, flavor and mealtime. This result was obtained from quality questionnaires regarding the service itself.

Flint et al. (27) evaluated the reproducibility, power and validity of the Blundell VAS (1982). As in our study, they compared the VAS score against single meal intake. All VAS questions were correlated with subsequent energy intake, which is partially in agreement with our observations. In our study, there was a negative correlation between the three questions of the EAA-BR and subsequent food consumption, that is, the lower the appetite, the greater the rest-ingestion index.

The scores of two questions (“How thirst do you feel?” and “How full do you feel?”) did not show a significant association with food acceptance or the rest-ingestion index. A possible explanation for our findings is that some patients reported drinking water prior to completing the questionnaire, which could have influenced their perception of thirst and difficulty in understanding the question “How full do you feel?”, which may have influenced patients’ response to the question. This paper had some limitations. First, only a small number of patients were evaluated. Second, the measure of rest-ingestion from only one daily meal (breakfast) can be considered as a bias and should be repeated with lunch and dinner.

It is of interest to carry out new studies with a greater number of patients and applying the EAA-BR to other meals and on subsequent days.

The EAA-BR appears to be sensitive for the assessment of appetite, presenting a significant correlation between questions about the food cravings, hunger, and prospective food consumption and dietary intake of cancer patients. The EAA-BR can be used routinely by health professionals for the quick and effective identification of the level of appetite of patients.

## CONCLUSION

The culturally adapted version of Blundel’s original English tool (1982) in Brazilian Portuguese (EAA-BR) demonstrated good validity compared with rest-ingestion measures when applied to cancer patients. This questionnaire could be a useful tool for evaluating appetite in Brazilian cancer patients in future studies.

## AUTHOR CONTRIBUTIONS

Ozório GA and de Almeida MMFA were responsible for the study design, data collection, data analysis and manuscript writing. Faria SO and Cardenas TC were responsible for the study design and manuscript revision. Waitzberg DL was responsible for the project drafting and critical revision of the manuscript for important intellectual content. All authors approved the final version of the manuscript and take public responsibility for the appropriate portions of the content.

## Figures and Tables

**Table 1:** Sociodemographic and clinical characteristics of the study sample.

	n	%
**Sex**		
Male	29	45.3
Female	35	54.7
**Age**		
Adult	43	67.2
Elderly	21	32.8
**Cancer type**		
Gastroenterology	14	21.9
Lung	4	6.3
Urology	14	21.9
Gynecology	19	29.7
Head and neck	4	6.3
Others	9	14.1
**Stage**		
II	14	21.9
III	8	12.5
IV	41	64.1
not applicable	1	1.6
**Main reasons for hospitalization**		
Treatment	24	37.5
Symptoms	18	28.1
Pain	13	20.3
Infection	4	6.3
Others	5	7.8
**Corticoids**		
Yes	20	31.3
No	44	68.8

**Table 2:** Nutritional characteristics of the patients.

	n	%
Nutritional screening* (NRS 2002)		
No nutritional risk	36	56.25
Nutritional risk	28	43.75
**Subjective global assessment (Desk 1987)**		
Nourished	16	57.1
Moderate malnourished	12	42.9
**Body mass index (BMI)**		
Low weight	7	10.9
Eutrophic	33	51.6
Overweight	12	18.8
Obesity	12	18.8

**Table 3:** Correlation between EAA-BR scores and rest-ingestion index of patients.

Breakfast	Median (DP)	RHO	*p*
Question 1	6.22 (2.71)	-0.3028	0.0046
Question 2	5.78 (2.92)	-0.2317	0.0319
Question 3	5.74 (2.79)	-0.0949	0.3847
Question 4	7.53 (2.42)	-0.3049	0.0043
Question 5	4.25 (2.53)	-0.0681	0.5335

Spearman’s test.
